# Successful partial-thickness skin grafting in a pediatric scalp avulsion after dog bite: A case report

**DOI:** 10.1016/j.ijscr.2024.110506

**Published:** 2024-10-23

**Authors:** Hamed Shahdadi, Fatemeh Shahrahmani, Somayeh Rezayi, Morteza Hashemian

**Affiliations:** a Department of Plastic and Reconstructive Surgery, Kerman University of Medical Sciences, Kerman, Iran; b Faculty of Medicine, Kerman University of Medical Sciences, Kerman, Iran; c Clinical Research Development Unit, Shafa Hospital, Kerman University of Medical Sciences, Kerman, Iran; d Department of Anesthesiology and Pain Medicine, Kerman University of Medical Sciences, Kerman, Iran

**Keywords:** Animal bites, Graft, Scalp defect, Partial-thickness skin graft, Case report

## Abstract

**Introduction:**

Animal bites can cause significant head and neck injuries in children with scalp avulsions posing a challenge. This report presents a case of successful partial-thickness skin grafting in managing a severe pediatric scalp avulsion from a dog bite.

**Case presentation:**

A 7-year-old boy who sustained extensive scalp injuries following a dog attack was admitted to the emergency room. The patient presented in hypovolemic shock with deep lacerations and full-thickness soft tissue avulsion involving the frontal, parietal, and occipital regions of the scalp. Initial treatment included fluid resuscitation, antimicrobial prophylaxis, and wound stabilization. Over a 45-day period, the patient underwent multiple debridements, resection of the outer cortex of the scalp bone, and preparation of the wound bed with a vacuum-assisted closure (VAC) device. Partial-thickness skin grafts (PTSGs) were harvested from the anterior thighs and successfully applied to the granulation tissue, resulting in a graft take rate exceeding 90 %. The patient was discharged in good condition with a satisfactory outcome.

**Discussion:**

The case underscores the efficacy of PTSGs in managing extensive scalp defects in pediatric patients. The technique offers several advantages, including quicker donor site healing, adaptability to irregular surfaces, and a high success rate in challenging wound beds. Compared to other reconstruction methods, PTSG is particularly beneficial when donor sites are limited.

**Conclusion:**

This case report highlights the successful use of PTSGs in the treatment of a severe pediatric scalp avulsion, demonstrating its viability as a reliable option for extensive scalp reconstruction in children.

## Highlights

• Successful use of partial-thickness skin grafts in pediatric scalp reconstruction.
• High graft take rate (>90 %) in a complex scalp injury from a dog bite.
• Favorable cosmetic results achieved with partial-thickness skin grafting method.

## 1 Introduction

Injuries to the head and neck region constitute a substantial proportion of animal dog bites, ranging from 26.8 % to 56.5 % [1]. In addition, 37.1 % of dog bites occurred among patients younger than 18 years. Due to their small stature, which is often comparable to the height of animals, children are particularly vulnerable to trauma in these areas [1,2].

Full-thickness and partial-thickness skin defects caused by animal bites present a significant challenge for reconstructive surgeons, particularly in the scalp region [3]. The scalp's tight skin and limited blood supply constrain the use of local tissue for primary closure or advancement flap, particularly in the case of extensive wounds. Immediate coverage of exposed bone is crucial to prevent infection and heat loss [4]. Addressing these defects requires a careful balance between restoring cranial protection and achieving a cosmetically acceptable outcome [5]. Restoring extensive scalp wounds in children presents unique challenges, as these injuries often exceed the size of available donor sites and are complicated by limited tissue resources, and involvement of hair-growing areas [6].

Treating scalp avulsion involves various surgical techniques. The preferred approach, when feasible, is microsurgical replantation [7]. If replantation is not possible, alternative methods include skin grafting, free or local flap reconstruction, and tissue expansion [[7], [8], [9]]. Skin grafting is an effective method for scalp reconstruction, particularly for large defects or when local flaps are not feasible [10]. Both split-thickness skin grafts, also known as partial-thickness skin grafts (PTSGs), and full-thickness skin grafts (FTSGs) can be utilized. PTSGs heal quickly, cause less pain, and result in more acceptable scarring [11].

In this study, we present the details of the application of PTSGs in a seven-year-old patient who sustained severe scalp injuries from a dog attack. The goal is to present an effective approach to managing complex pediatric scalp traumas.

This manuscript was prepared following the SCARE guidelines [12].

## 2 Case report

**Fig. 1:**
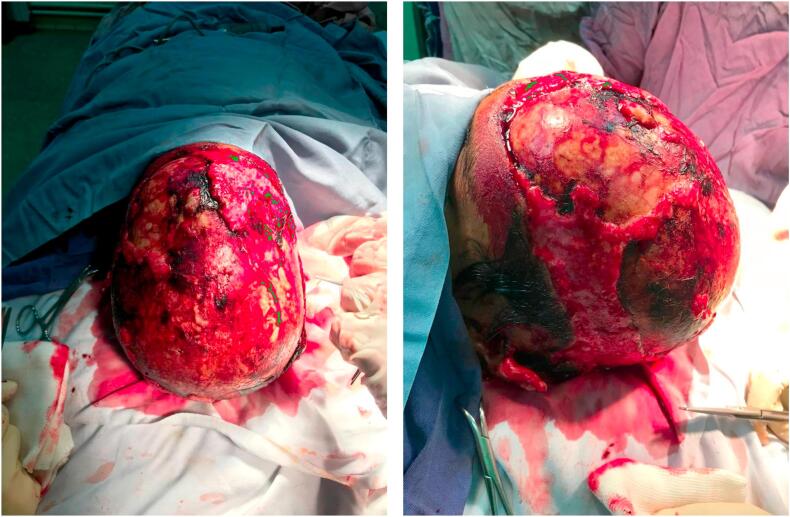
Extensive scalp injuries.

The patient was a 7-year-old boy who presented to the emergency department following an attack by a dog. Upon arrival, he exhibited signs of hypovolemic shock, including tachycardia (pulse rate of 130 beats per minute) and hypotension (systolic blood pressure of 70 mmHg). The initial clinical assessment revealed multiple deep lacerations to the scalp, along with numerous wounds distributed across the craniofacial and cervical regions. A central venous catheter was inserted to facilitate fluid resuscitation and blood product administration until the patient achieved hemodynamic stability. Antimicrobial prophylaxis was initiated with a combination of cefazolin and clindamycin. He was administered tetanus prophylaxis and rabies post-exposure prophylaxis, including rabies immunoglobulin and vaccine, according to established protocols. Further examination indicated full-thickness soft tissue avulsion involving the scalp's frontal, parietal, and occipital regions. The patient also sustained partial tearing and avulsion injuries to both ears. The scalp and facial injuries were provisionally dressed to maintain a sterile environment and promote optimal wound healing. Following the patient's stabilization, he was taken to the operating room for a detailed examination of the wounds under anesthesia. A substantial defect measuring approximately 10 × 30 cm was identified in the frontal region, along with two large wounds on both sides of the parietal lobes. Additionally, the occipital region presented a significant defect, approximately 10 × 10 cm (Fig. 1). The wounds were irrigated and dressed with Vaseline petrolatum gauze.

After 72 h, the patient was returned to the operating room for a targeted procedure to address the severe scalp injuries. The surgical intervention involved the complete excision of the full thickness of the scalp, including the skin, underlying soft tissues, and periosteum in the affected areas. This surgery focused on meticulous debridement of the wound sites, aiming to remove any non-viable tissue and optimize the conditions for subsequent healing and potential reconstructive interventions.

**Fig. 2:**
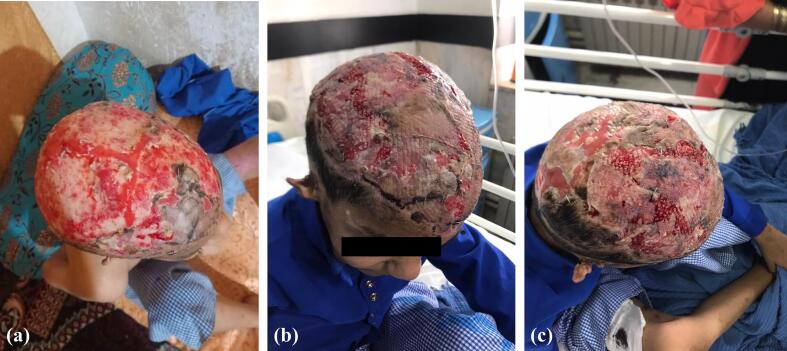
Full granulation tissue before PTSGs (a) and PTSGs after seven days (b, c).

Resection of the outer cortex of the scalp bone down to the spongy layer was performed in four stages, spaced five days apart, to promote the development of sufficient granulation tissue for grafting. A vacuum-assisted closure (VAC) device was applied to aid healing. The VAC dressing was used over the course of 45 days. Once adequate granulation tissue had formed (Fig. 2), the vacuum dressing was removed, and the graft was performed three days later. PTSGs were harvested from the anterior aspects of both thighs in two sessions, spaced three weeks apart, and were applied to the granulation tissue at the defect site.

**Fig. 3:**
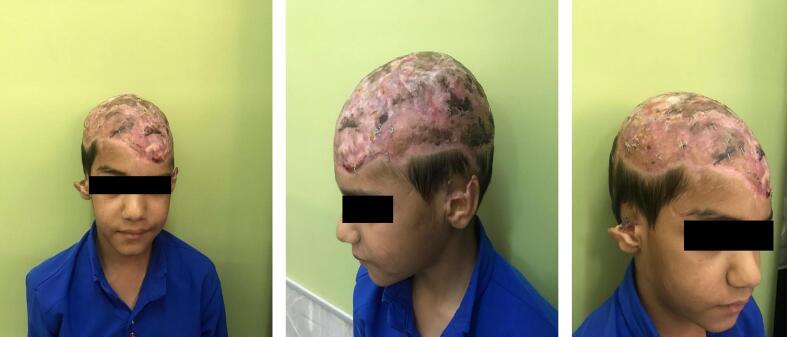
PTSGs grafts after one months.

**Fig. 4:**
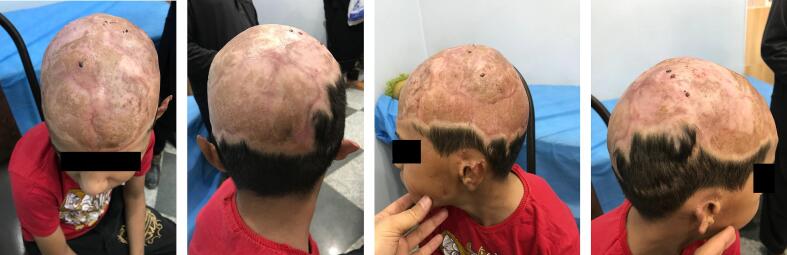
PTSGs after four months.

**Fig. 5:**
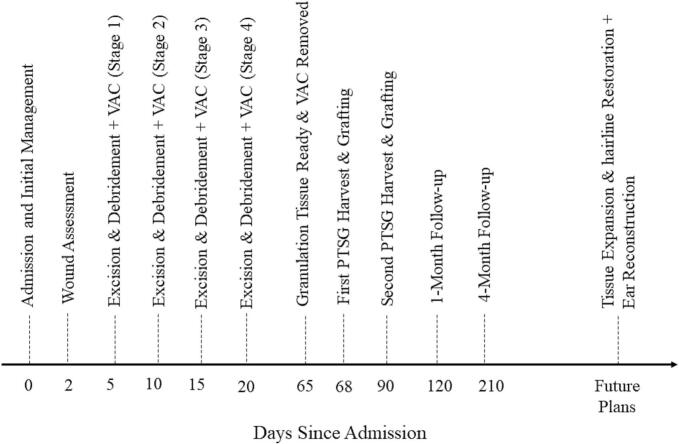
Schematic Timeline of Patient Management.

After seven days, the dressings were removed, revealing a graft take rate exceeding 90 %, indicating successful integration of the grafted tissue (Fig. 2). The patient was discharged in good general condition and frequent followed up exams were performed. In follow-ups, at approximately one (Fig. 3) and four (Fig. 4) months after the surgery, the condition of the grafts was excellent, and the outcome was highly satisfactory. Fig. 5 provides a timeline of patient management.

For future hairline restoration, tissue expansion will be employed on the remaining hair-bearing scalp to stretch healthy tissue and provide additional coverage. Once sufficient expansion is achieved, the affected grafts will be excised, and the expanded skin repositioned to restore a natural hairline, ensuring both aesthetic and functional improvements. The patient also sustained partial tearing and avulsion of both ears. Given the case's complexity, ear reconstruction was deferred until tissue expansion is complete. Future reconstruction will involve staged procedures using autologous rib cartilage to restore ear structure.

## 3 Discussion

A significant percentage of children experience animal bites during childhood, with the head and neck areas being particularly vulnerable [1]. Pediatric scalp avulsion injuries caused by dog bites are rare but present significant challenges for reconstructive surgeons [2]. Treating scalp avulsion involves various surgical techniques, with the choice of restoration method depending on factors such as the extent and location of the injury, as well as the surgeon's expertise [13]. The preferred approach, when feasible, is microsurgical replantation because of its ability to restore both function and aesthetics. If replantation is not viable due to the extent of damage or necrosis, alternative reconstructive methods become necessary [9]. For small or medium-sized defects, local or regional flaps may be used to preserve native tissue and hair-bearing skin. When local tissue is unavailable, free flaps, which involve transferring vascularized tissue from a distant donor site, are another option [5]. Additionally, in cases involving extensive tissue loss or scarring, tissue expansion may be employed to generate additional scalp skin over time. While tissue expansion provides hair-bearing skin, the process requires multiple surgeries over several months, making it a longer, staged treatment option [2].

Skin grafts offer a valuable alternative when primary closure is not feasible [14]. Skin grafts, especially PTSGs, offer several advantages over other reconstruction techniques for animal bite injuries. They demonstrate high success rates on different surfaces, including dermis, granulation tissue, fat, and fascia [15]. Skin grafts can cover larger recipient areas compared to primary repair or local flaps, making them particularly beneficial for extensive injuries with significant tissue loss [16]. PTSGs have lower metabolic demands, which can potentially increase survival rates in challenging wound beds, and they can adapt more easily to the irregular surfaces often seen in bite wounds. Additionally, they allow for quicker donor site healing with less scarring [17,18].

In this case, due to the severity of the injury and the extent of tissue loss caused by the dog attack, replantation was deemed unfeasible. Although using a dermal matrix like Integra®, followed by tissue expansion, could have been an ideal strategy, these materials were unavailable in our setting and posed an increased risk of infection. Free flap reconstruction, with the superficial temporal artery as the recipient vessel, was also considered. However, given the complexity of free flap surgery in pediatric patients and the need to minimize both intraoperative risks and recovery time, a PTSG was selected. This approach enabled timely wound closure with a favorable outcome.

To date, few studies have suggested skin graft as a reliable and cosmetically satisfactory method for management of complex scalp defects. A noteworthy case reported by Konofaos et al. [19] described the successful reconstruction of a large full-thickness scalp defect in a 2-year-old patient following a dog attack. The technique involved the application of a cryopreserved human skin allograft for initial wound bed preparation, followed by the use of Integra, a dermal regeneration template, which was applied after burring out the outer bony cortex to enhance integration. Subsequent grafting with split-thickness skin grafts resulted in a 98 % take rate and a stable, pliable soft-tissue cover one year post-procedure.

Bašković et al. [20] demonstrated the successful use of a combined approach involving the VAC system and the Integra® dermal regeneration template in a one-year-old boy with a massive scalp injury caused by a dog bite. After the initial reimplantation of scalp tissue failed, a necrectomy was performed, and the wound was prepared using the VAC system. Following this, the application of Integra® led to successful wound integration, allowing for subsequent split-thickness skin grafting with a favorable outcome.

These results may support the use of PTSG as a viable option for treating complex scalp injuries. The success of this technique after animal bites is generally high when performed by experienced surgeons and combined with appropriate wound management techniques.

## 4 Conclusion

This case report highlights the successful management of a complex pediatric scalp avulsion injury due to a dog bite through the application of PTSG. The outcome, marked by a graft take rate exceeding 90 % and a highly satisfactory cosmetic result at the four-month follow-up, underscores the viability of PTSG as a reliable technique for extensive scalp reconstruction in pediatric patients. This case adds to the growing body of research supporting the use of PTSGs in managing severe scalp injuries, particularly when other reconstructive options are not feasible.

## Patient consent

Written, informed consent was obtained from the guardian of the participating patient for the publication of anonymized information in this article.

## Ethical approval

Ethical approval was obtained from the ethics review board.

## Declaration of Generative AI and AI-assisted technologies in the writing process

During the preparation of this work, the authors did not utilize any generative AI or AI-assisted technologies.

## Sources of funding

The authors declare that there was no direct or indirect financial support by extramural sources for the study.

## Declaration of competing interest

The authors declare that they have no related conflicts of interest to this work.
